# Dr. Karunakarnta Goswami

**Published:** 2011

**Figure:**
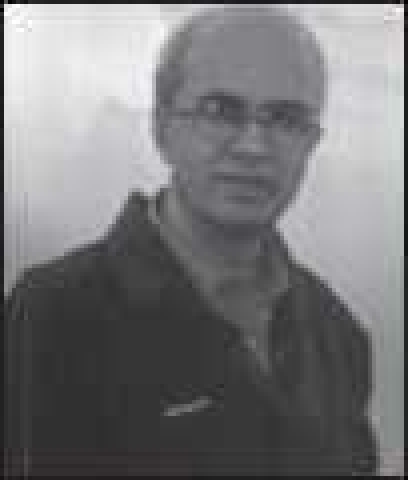
(2^nd^ Jun 1947 – 21^st^ Sept 2010)

Dr. Karunakarnta Goswami, born on 2 June 1947, was as active member of I.S.A. We regret to inform the sad demise of Dr. Goswami who passed away on 21^st^September 2010.

Dr. Goswami was the Vice-President of Guwahati City Branch of I.S.A. at the time of his death. He did M.D. (Anaesthesiology) in 1986 from Guwahati Medical College and after that was engaged in freelance practice and established himself as a renowned anaesthesiologist of Guwahati. He took special interest in pain management and was managing his own acupuncture clinic. Dr. Goswami was very regular in attending national conference of I.S.A. He left behind his wife Dr. Iva Goswami, a prominent Gynaecologist, a son and daughter. Guwahati City Branch of I.S.A. deeply mourns the untimely demise of Dr. Goswami.

